# Supplementary material for: 4‐Thiaproline accelerates the slow folding phase of proteins containing cis prolines in the native state by two orders of magnitude
Source: Protein Sci. 2024 Feb 1;33(2):e4877. doi: 10.1002/pro.4877 (PMC10804670; doi:10.1002/pro.4877)
Supplement: Supplementary file 1 — Data S1. Supporting Information [file PRO-33-e4877-s001.pdf]

Supporting Information

4-Thiaproline accelerates the slow folding phase of proteins containing *cis* prolines in the native state by two orders of magnitude

Jennie O' Loughlin,[1] Kirill Zinovjev,[2,3] Silvia Napolitano,[4] Marc van der Kamp,[2] and Marina Rubini*[1]

[1] School of Chemistry, University College Dublin, Belfield, Dublin 4, Ireland

E-mail: marina.rubini@ucd.ie

[2] School of Biochemistry, University of Bristol, UK

[3] Department of Physical Chemistry, University of Valencia, Spain

[4] Department of Molecular Biology and Biophysics, ETH Zürich, 8093 Zürich, Switzerland

1. Materials

1.1 Determination of the redox potential of Trx1Thp

2. Supplementary Figures

3. Supplementary Tables

4. References

1. Materials

The following were obtained from Sigma: NADPH tetrasodium salt, 5,5'-dithiobis-2-nitrobenzoic acid (DTNB), insulin from bovine pancreas. L-Thiazolidine-4-carboxylic acid (98% purity) was purchased from Alfa Aesar. *E. coli* TrxR was a gift from Prof. Rudi Glockshuber (ETH Zurich).

1.1 Determination of the redox potential of Trx1Thp

Redox potentials were determined via their equilibrium constant, using Trx wt as a reference, following published procedures.[1] First, Trx wt was freshly reduced in 1 mM DTT and subsequently all Trx variants were buffer exchanged against 50 mM MOPS·NaOH pH 7.0, 1 mM EDTA using PD MiniTrap G-25 columns (GE Healthcare) (500 µL protein load, 200 µL wash and 700 µL elution volume). Oxidized Trx variants were mixed with reduced Trx wt in a final volume of 100 µL (Trx variant 10 µM, Trx wt 10-20 µM), and incubated for 17 hours at 25°C to attain equilibrium. The disulfide exchange reaction was quenched by addition of 12 µL formic acid and 50 µL were loaded on a ZORBAX C8 reverse phase column (300 Å, 4.6 x 250 mm, from Agilent), equilibrated with 35% acetonitrile in water, 0.1% TFA. Protein were eluted with a gradient from 35 to 65% acetonitrile in water, 0.1% TFA over 30 min, with a flow rate of 1 mL/min and a temperature of 70°C. Elution profiles were recorded following the absorbance signal at 220 nm, and protein concentrations calculated via their peak area (using the Peak Analyzer tool of OriginPro 2018b, OriginLab). Redox equilibrium constants were calculated according to Equation 1 and obtained K_{eq} were used to determine the redox potential (E'_0) of each Trx variant, according to Nernst equation (Equation 2), using E'_0 of the Trx wt_{red}/Trx wt_{ox} (-270 mV) redox couple as a reference.[2]

Equation 1: $K_{eq} = \frac{[Trx\ variant_{red}][Trx\ wt_{ox}]}{[Trx\ variant_{ox}][Trx\ wt_{red}]}$

Equation 2: $E'_0 = -270\ mV + (\frac{RT}{2F} lnK_{eq})$

2. Supplementary Figures

Supplementary figure 1

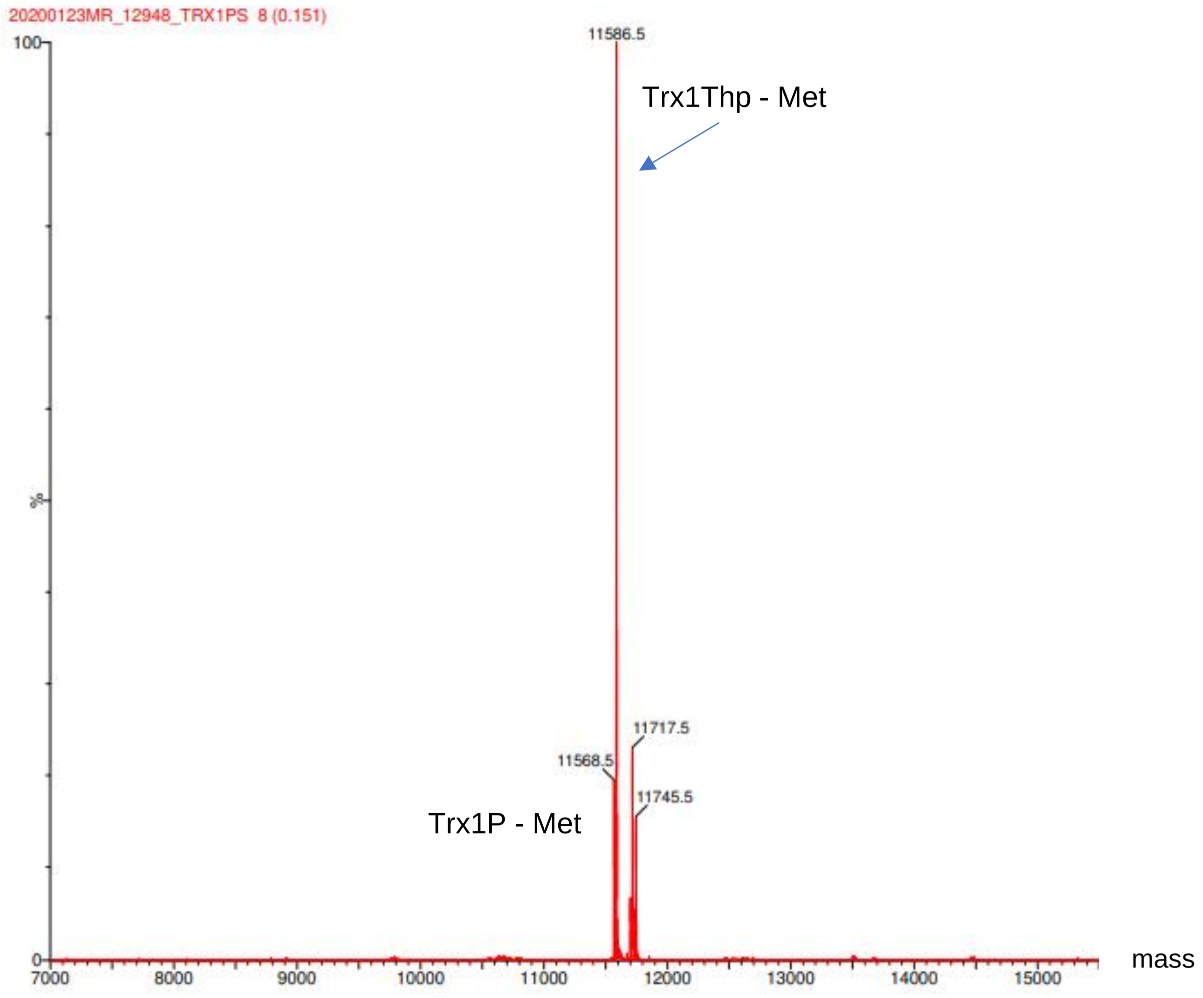

Supplementary Figure 1. Deconvoluted ESI-MS spectrum of *E. coli* Thioredoxin variant Trx1Thp.

Supplementary figure 2

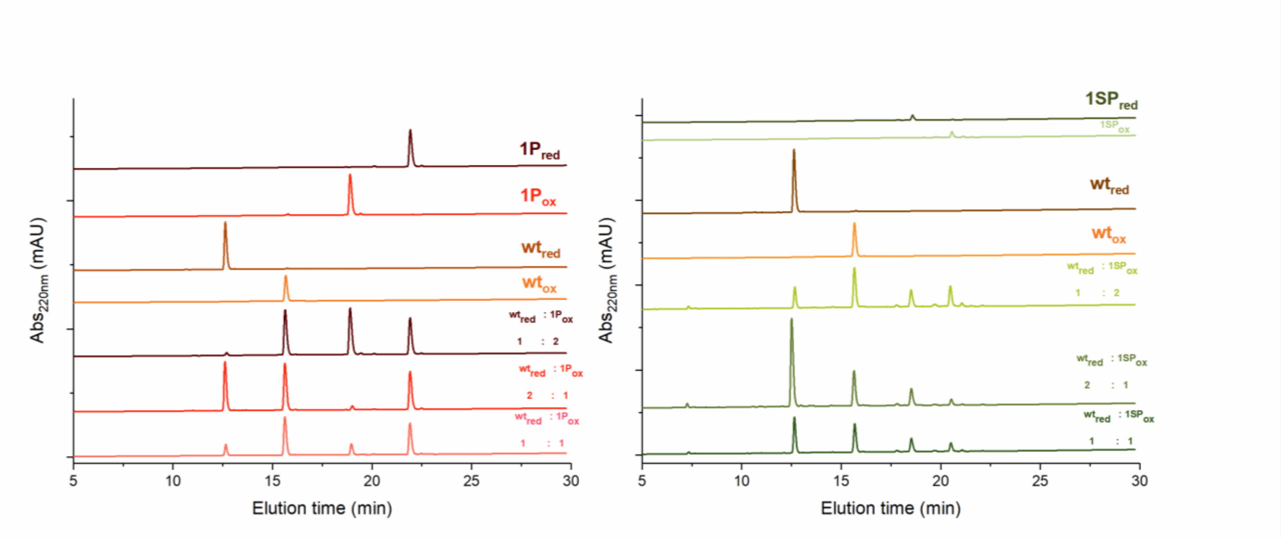

Supplementary Figure 2. Redox equilibria between Trx wt and Trx1P or Trx1Thp (1SP) at pH 7.0 and 25°C. Reduced Trx wt was mixed with the oxidized form of the respective Trx variant at a 1:1, 1:2 or 2:1 molar ratio. The reactions were quenched with formic acid, and all redox forms separated by reversed phase HPLC. Peak areas were converted to concentrations, from which redox equilibrium constants (K_{eq}) were calculated. K_{eq} values were found to be independent of the mixing ratio between reduced Trx wt and oxidized Trx1P or Trx1Thp, showing that the redox equilibria were attained.

Supplementary figure 3

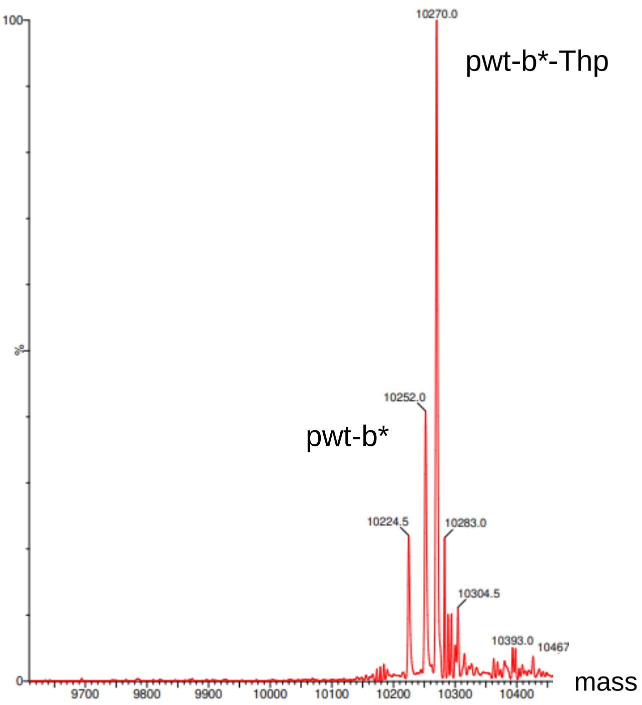

Supplementary Figure 3. Deconvoluted ESI-MS spectrum of *B. amiloliquefaciensi* barstar variant pwt-b*-Thp (calculated: 10270.6 Da; found: 10270.0 Da).

3. Supplementary Tables

Table S1. $K_{trans/cis}$ values, rate constants of *cis*/*trans* isomerization, and activation energies for *cis*/*trans* isomerization of model compounds.

**Compound**	$K_{trans/cis}$[a]	$k_{cis/trans}$ (s^{-1})	**ΔG‡ (kJ/mol)**	**Ref**
Ac-Pro-OMe	4.6			[4]
Ac-Thp-OMe	2.8			[5]
Ala-Pro-(4-)nitroanilide		0.055 ± 0.005	80.7 ± 0.3	[6]
Ala-Thp-(4-)nitroanilide		4.0 ± 1	70.0 ± 0.6	[6]
Ala-Gly-Pro-Phe-(4-)nitroanilide		0.036 ± 0.005	81.8 ± 0.3	[6]
Ala-Gly-Thp-Phe-(4-)nitroanilide		4.0 ± 1	70.0 ± 0.7	[6]

Table S2. Structural properties of the DFT (M06-2X/cc-pVTZ) optimized reactant and transition states for the different peptide bond rotations.

	**'Pro'**			**'Thp'**		
	q_m (Å)[a]	ϕ_m (°)[a]	ω (°)[b]	q_m (Å)[a]	ϕ_m (°)[a]	ω (°)[b]
**Reactant**	0.385	16.6	179.0	0.474	164.0	176.4
**TS 1↑**	0.432	57.3	–60.3	0.345	87.6	–63.3
**TS 2↑**	0.426	58.0	120.3	0.377	97.7	118.9
**TS 1↓**	0.417	17.8	–119.1	0.462	88.3	–119.8
**TS 2↓**	0.410	21.6	61.4	0.454	90.8	64.0

[a] Ring puckering amplitude q_m and phase angle ϕ_m were calculated as described by Cremer & Pople (Eqs. 12 and 13 in that work).[3] [b] The dihedral angle describing rotation around the peptide bond.

4. References

[1] S. Napolitano, R. J. Reber, M. Rubini, R. Glockshuber, *Journal of Biological Chemistry* **2019**, *294*, 14105-14118.

[2] a) E. C. Moore, P. Reichard, *Journal of Biological Chemistry* **1964**, *239*, 3453-3456; b) A. P. Carvalho, P. A. Fernandes, M. J. Ramos, *Prog Biophys Mol Bio* **2006**, *91*, 229-248

[3] D. Cremer and J. A. People, *Journal of the American Chemical Society* **1975**, *97*, 1354–1358

[4] A. Choudhary, D. Gandla, G. R. Krow, R.T. Raines, *Journal of the American Chemical Society* **2009**, *131*, 7244-7246

[5] A. Choudhary, K. H. Pua, R. T. Raines, *Amino Acids* **2011**, *41*, 181-186

[6] D. Kern, M. Schutkowski, T. Drakenberg, *Journal of the American Chemical Society* **1997**, *119*, 8403-8408
